# Supplementary material for: Early Improvements in Disease Activity Indices Predict Long-Term Clinical Remission Suggested by the Treat-to-Target Strategy in Patients with Ankylosing Spondylitis Receiving TNF-α Inhibitor Treatment
Source: J Clin Med. 2021 Sep 21;10(18):4279. doi: 10.3390/jcm10184279 (PMC8469764; doi:10.3390/jcm10184279)
Supplement: Supplementary file 1 [file jcm-10-04279-s001.zip › Supplemental_Table_S1_J Clin Med.pdf]

Table S1 Response to treatment with tumor necrosis factor-α inhibitor as measured by the criteria of the Ankylosing Spondylitis Disease Activity Score (ASDAS), low Bath Ankylosing Spondylitis Disease Activity Index with normal C-reactive protein level (BASDAI-CRP), and at least 50% improvement in the Bath Ankylosing Spondylitis Disease Activity Index ($BASDAI_{50}$).

	3 months	9 months	15 months	21 months	27 months	33 months
**ASDAS-ID**	32.4 (44/136)	46.8 (59/126)	50.8 (60/118)	55.8 (63/113)	55.4 (62/112)	68.9 (71/103)
**ASDAS-MI**	55.3 (73/132)	66.4 (81/122)	69.0 (78/113)	70.4 (76/108)	73.8 (79/107)	75.5 (74/98)
**ASDAS-CII**	88.6 (117/132)	95.9 (117/122)	95.6 (108/113)	95.4 (103/108)	96.3 (103/107)	96.9 (95/98)
**BASDAI-CRP**	39.9 (55/138)	58.7 (74/126)	65.3 (77/118)	69.0 (78/113)	70.5 (79/112)	75.2 (79/105)
**$BASDAI_{50}$**	76.3 (106/139)	92.2 (118/128)	94.2 (113/120)	93.8 (106/113)	93.8 (105/112)	98.1 (103/105)

ASDAS-ID, Ankylosing Spondylitis Disease Activity Score Inactive Disease; ASDAS-MI, Ankylosing Spondylitis Disease Activity Score major improvement; ASDAS-CII, Ankylosing Spondylitis Disease Activity Score clinically important improvement; BASDAI-CRP, low BASDAI and normal CRP level; $BASDAI_{50}$, 50% improvement in BASDAI.
