# Supplementary figures and images for: Early Improvements in Disease Activity Indices Predict Long-Term Clinical Remission Suggested by the Treat-to-Target Strategy in Patients with Ankylosing Spondylitis Receiving TNF-α Inhibitor Treatment
Source: J Clin Med. 2021 Sep 21;10(18):4279. doi: 10.3390/jcm10184279 (PMC8469764; doi:10.3390/jcm10184279)

**A**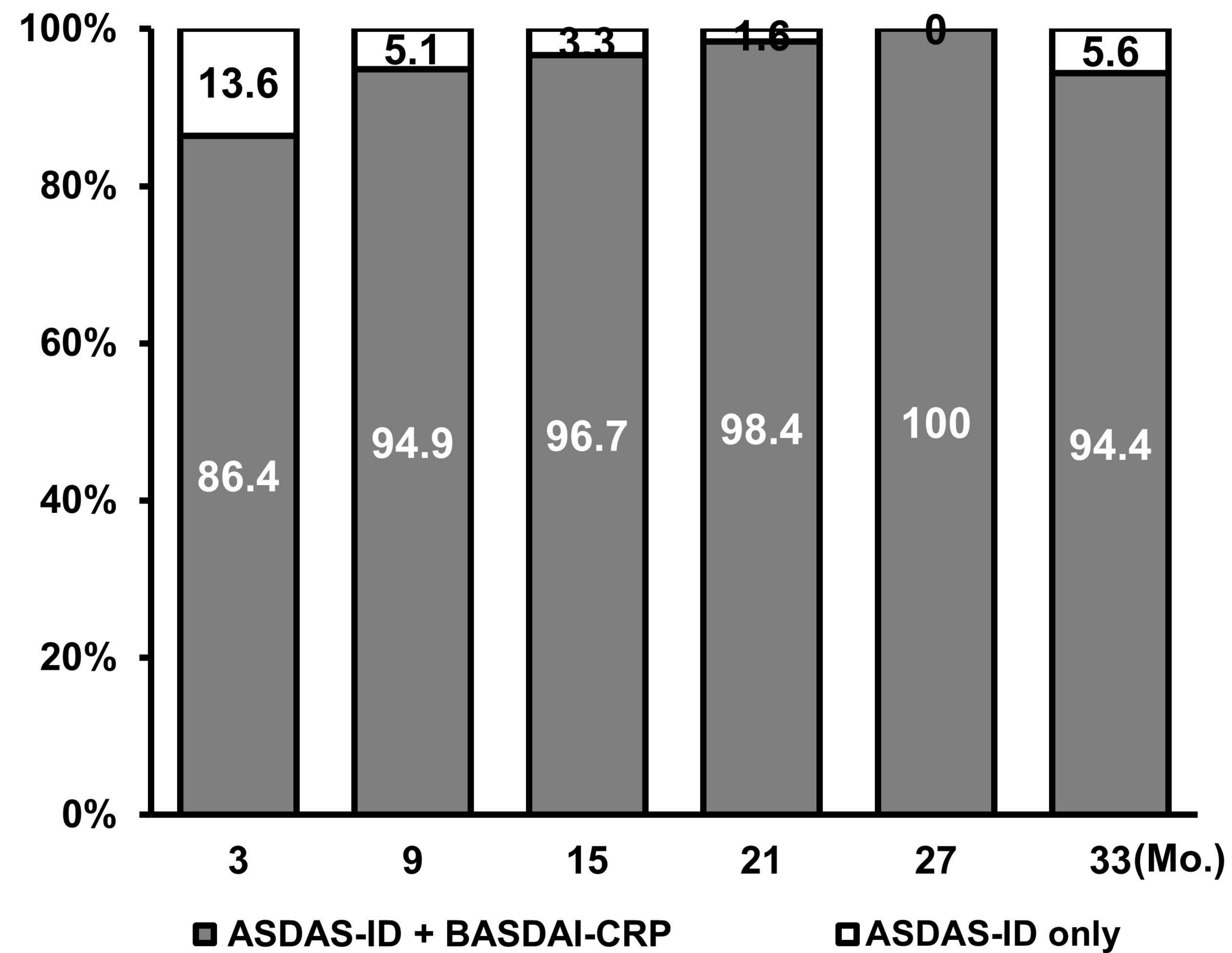**B**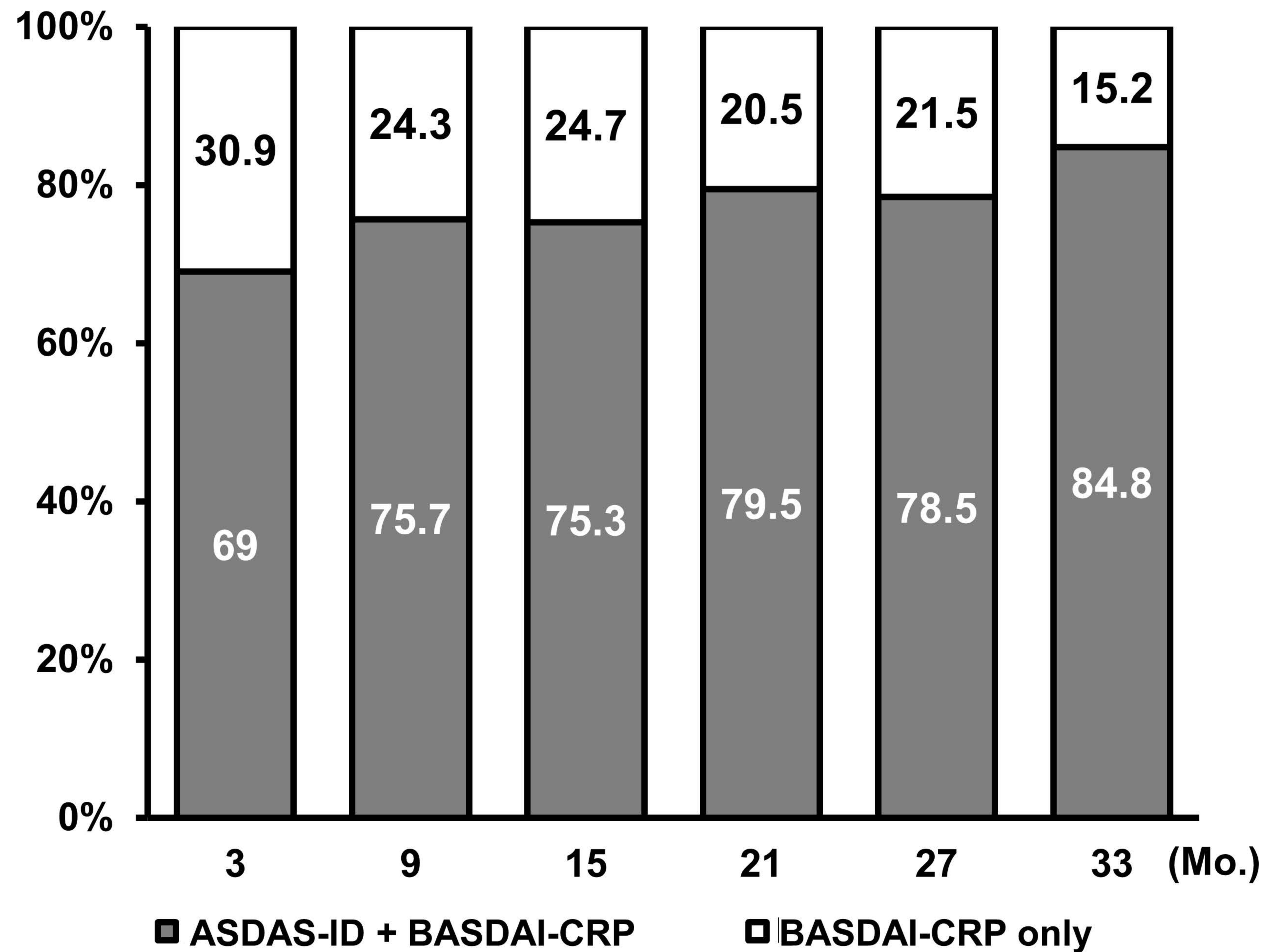

Supplement: Supplementary file 1 [file jcm-10-04279-s001.zip › Figure S1-J Clin Med.pdf]

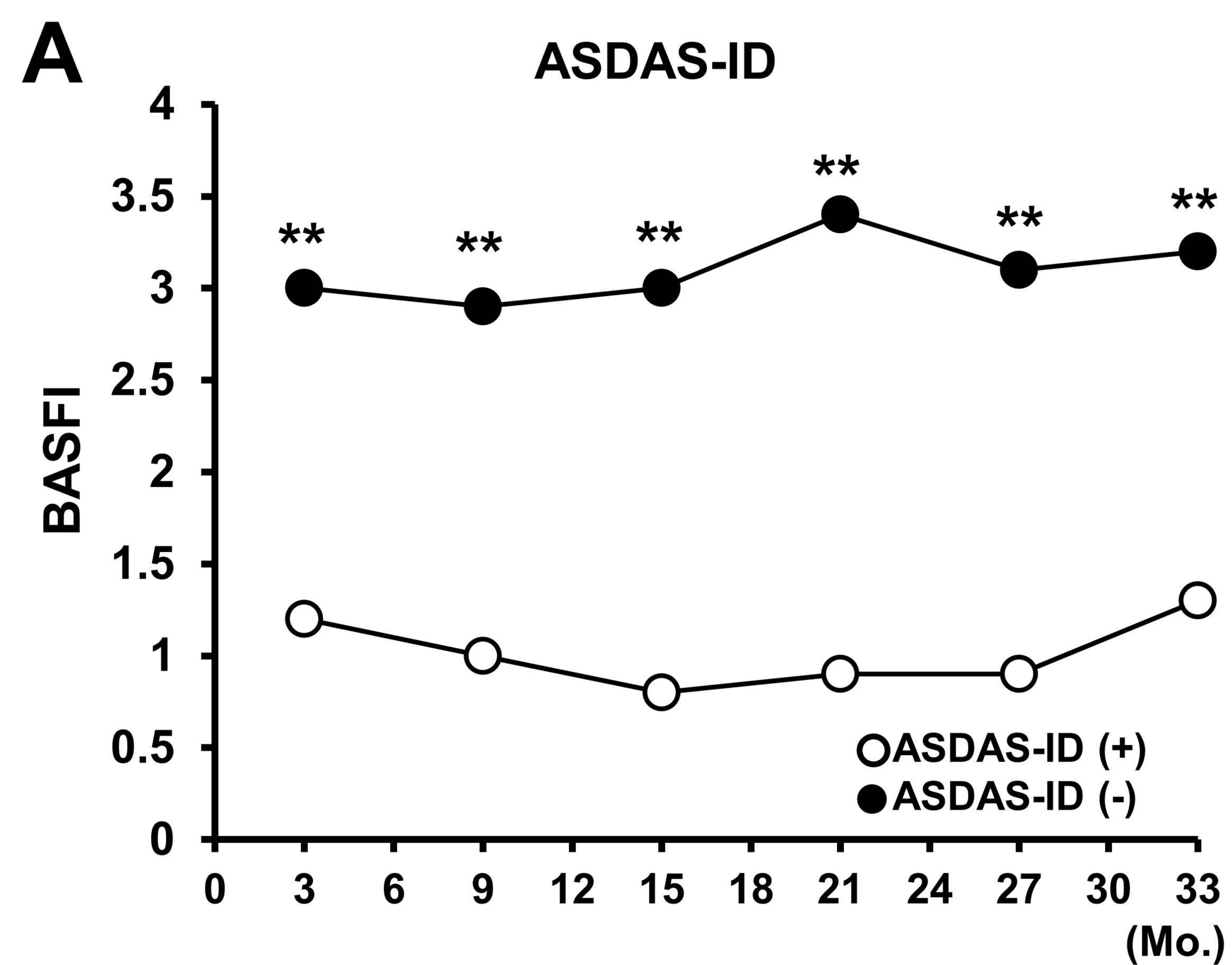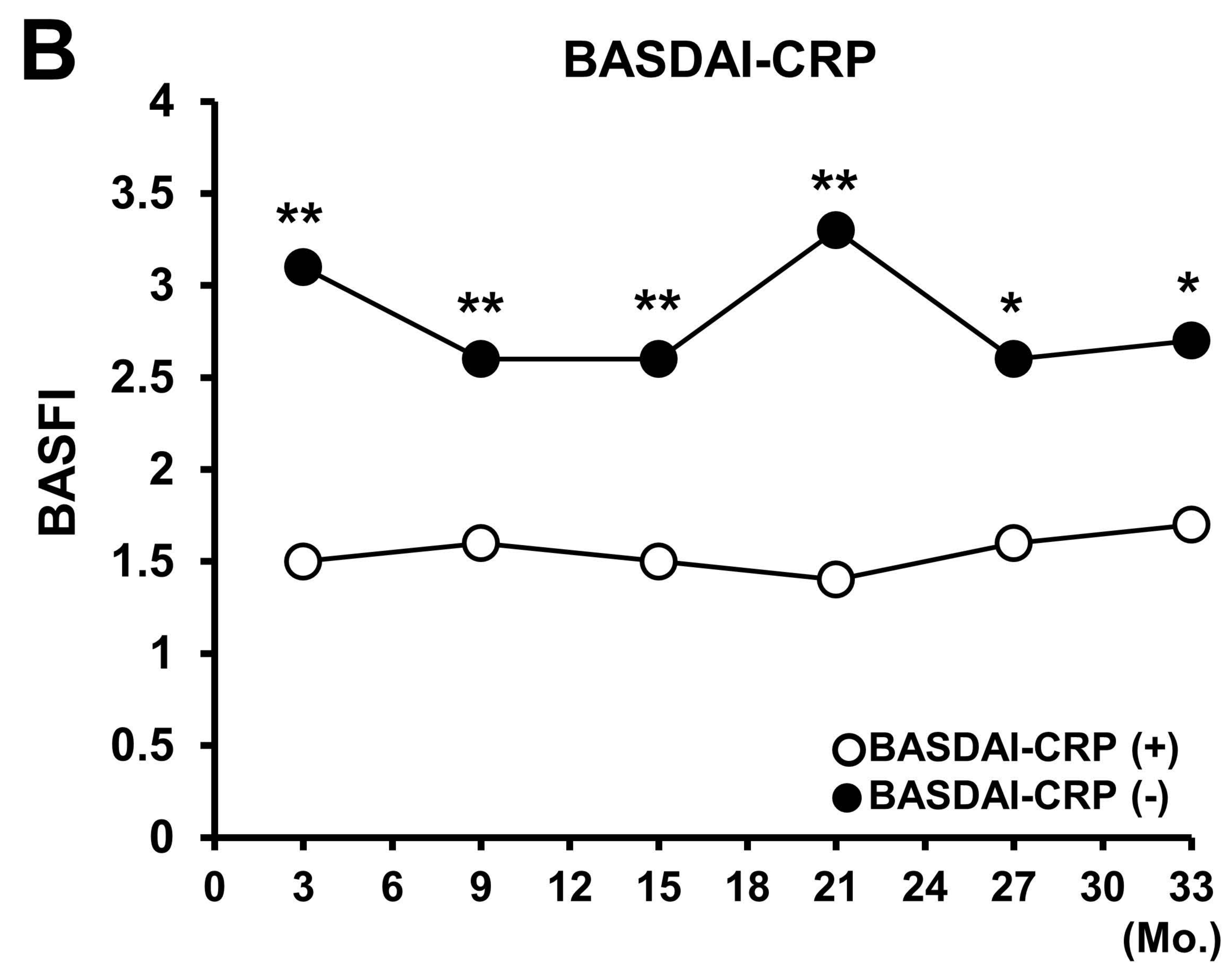

Supplement: Supplementary file 1 [file jcm-10-04279-s001.zip › Figure S2-J Clin Med.pdf]

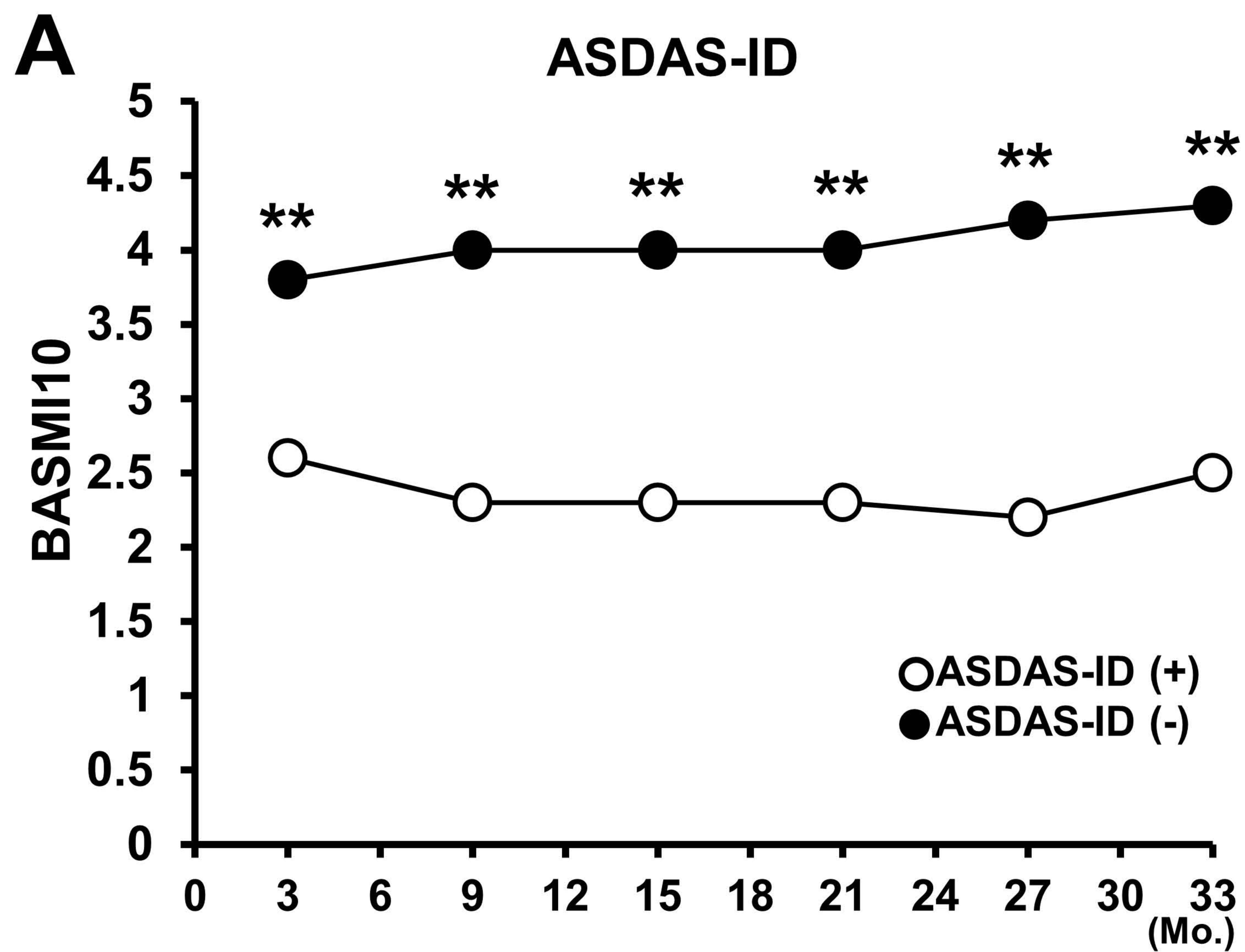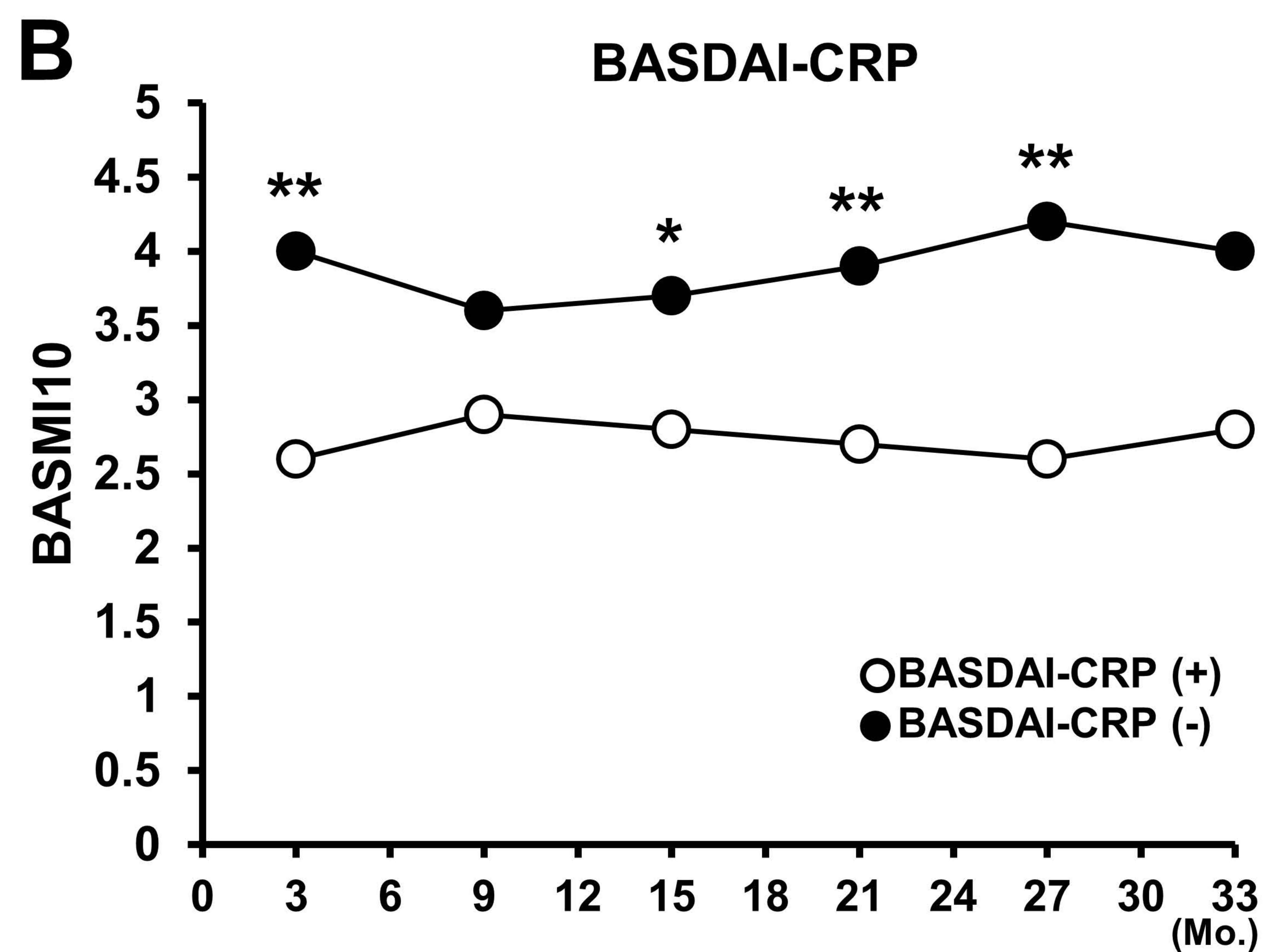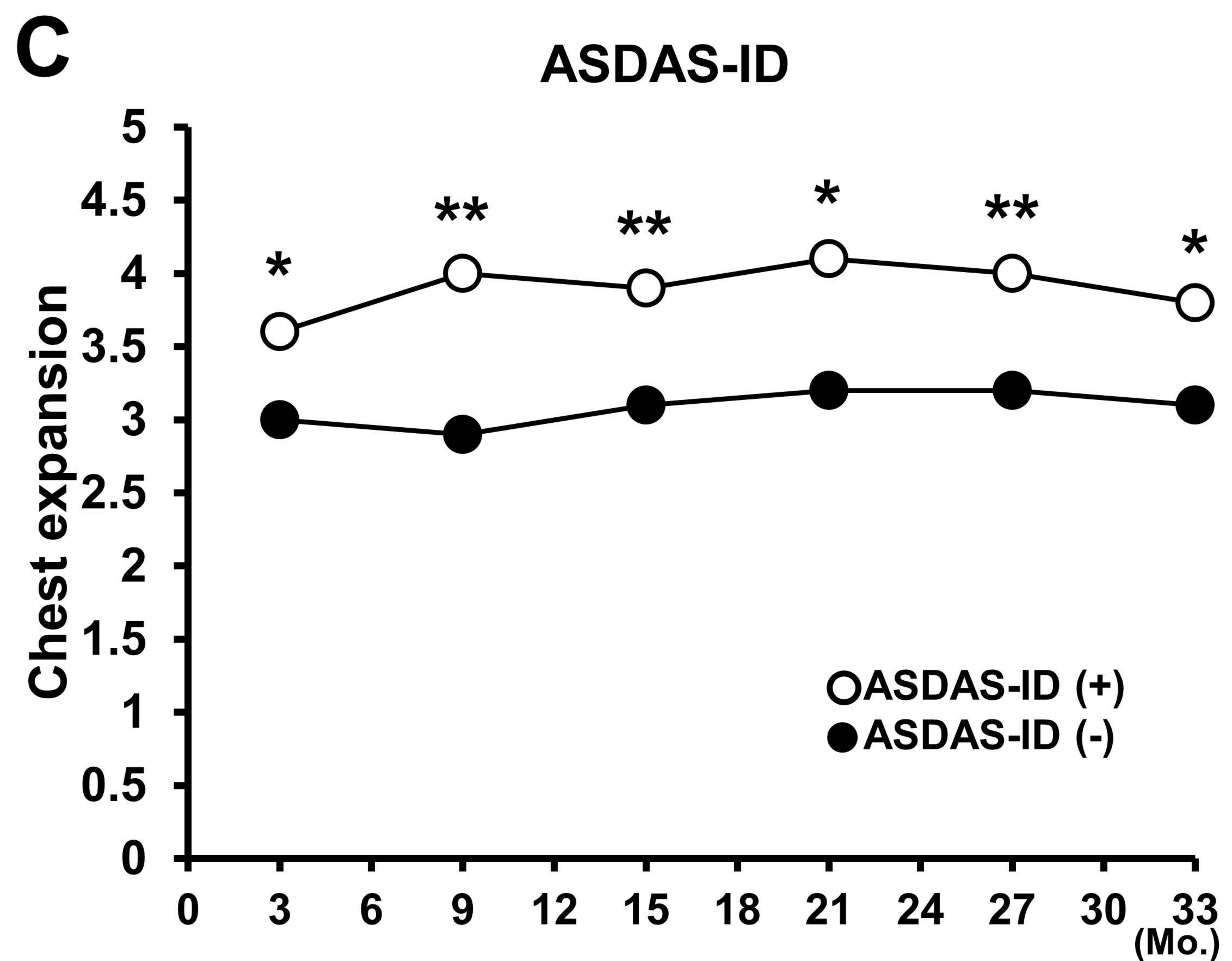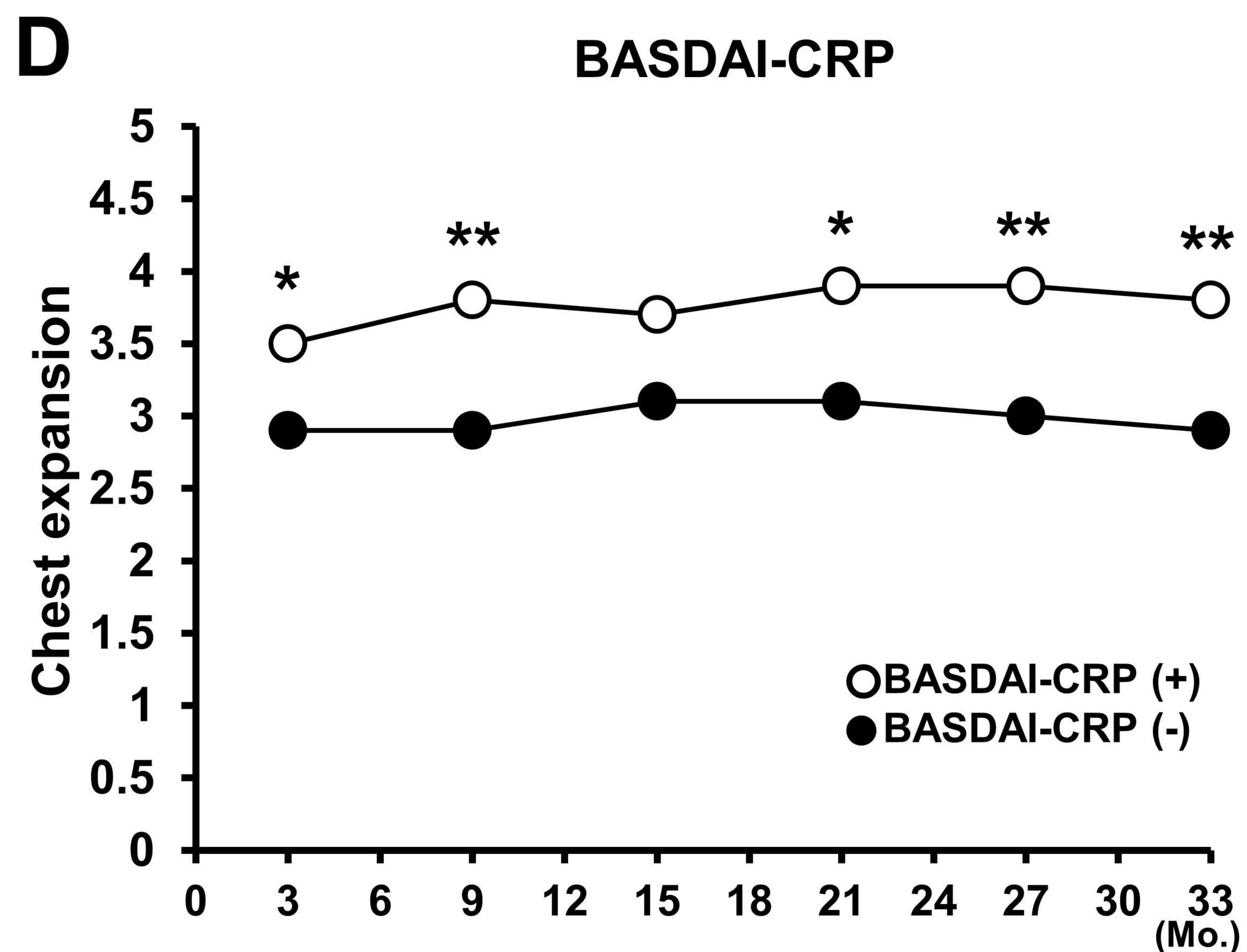

Supplement: Supplementary file 1 [file jcm-10-04279-s001.zip › Figure S3-J Clin Med.pdf]

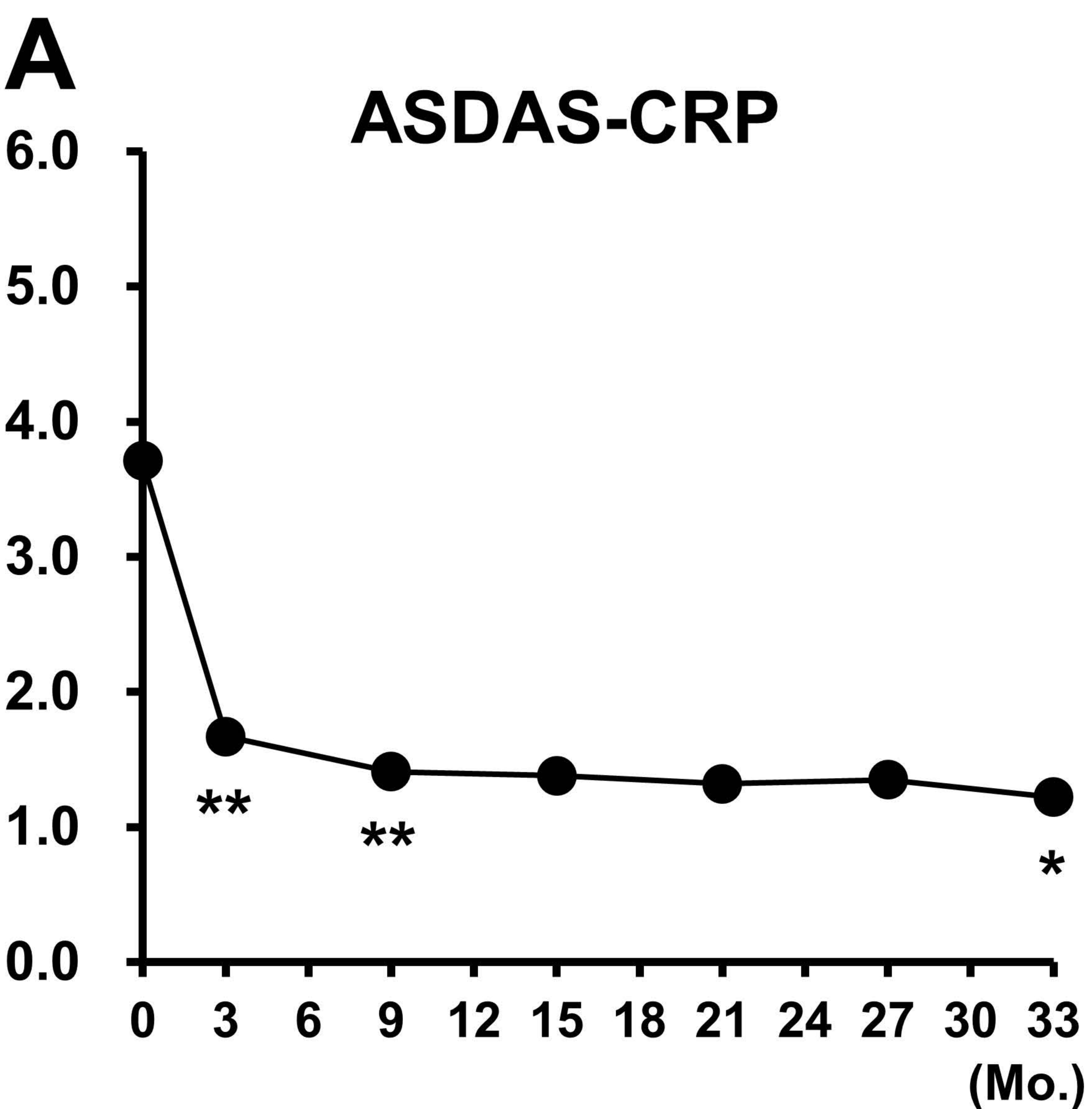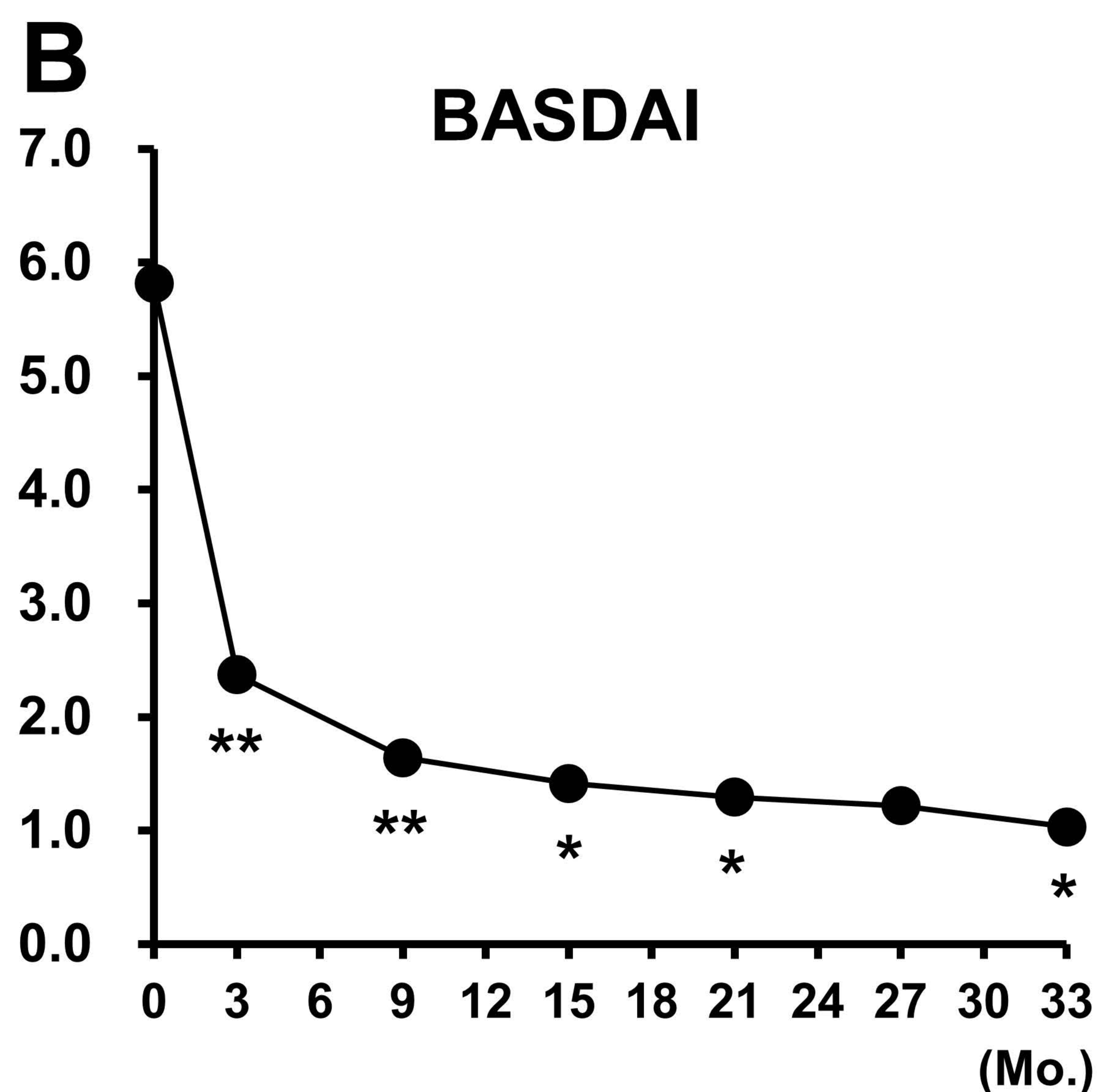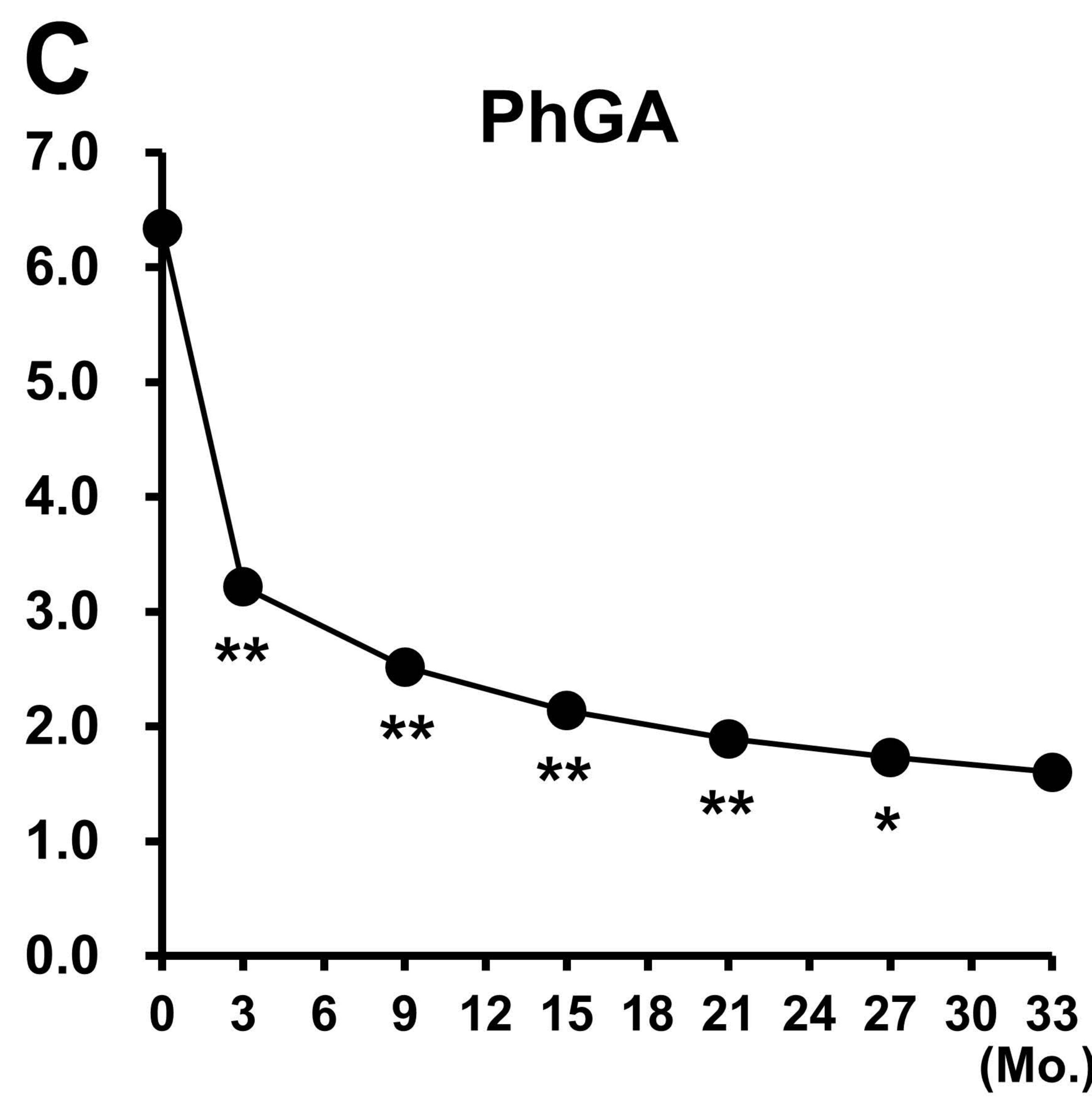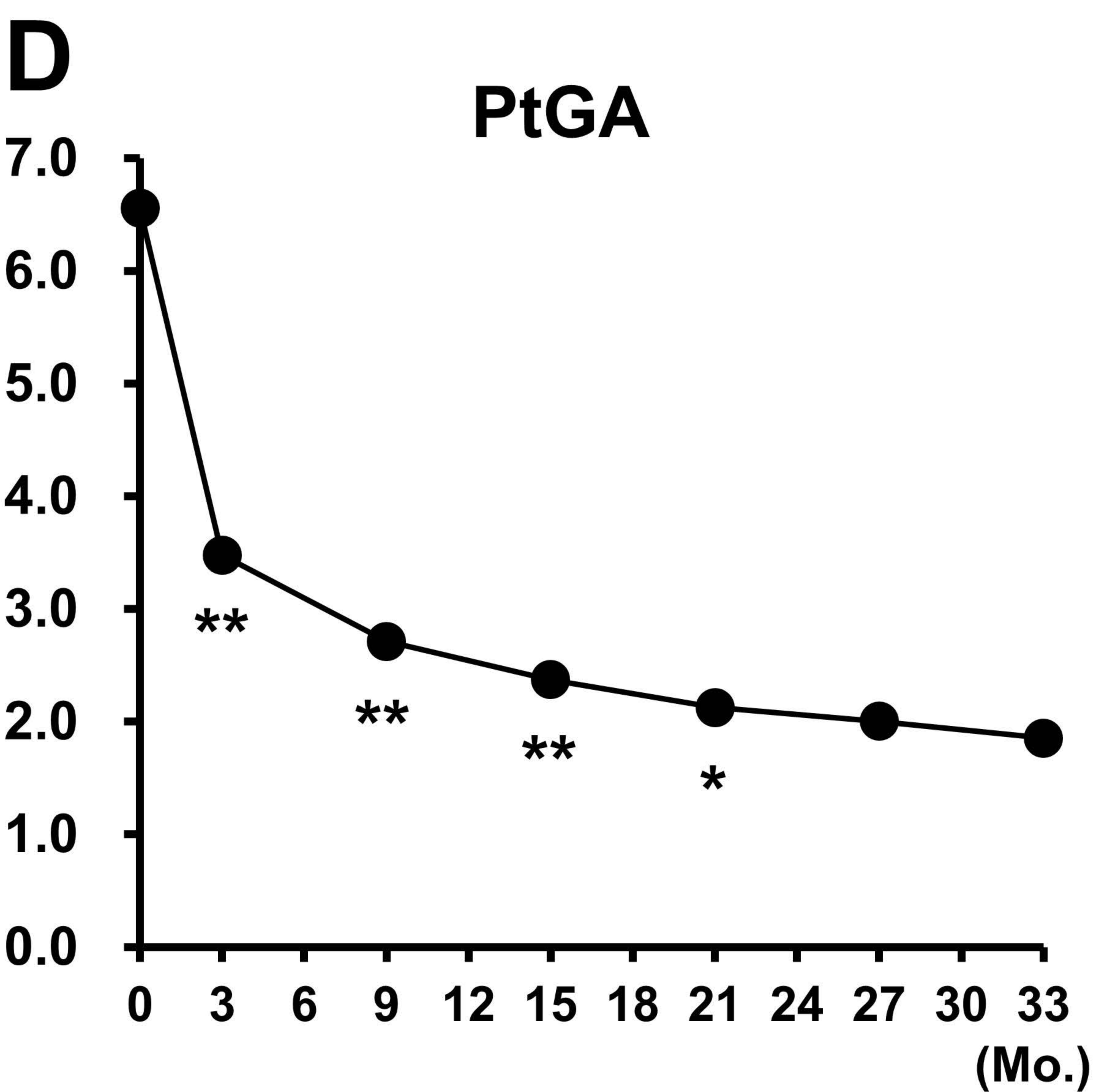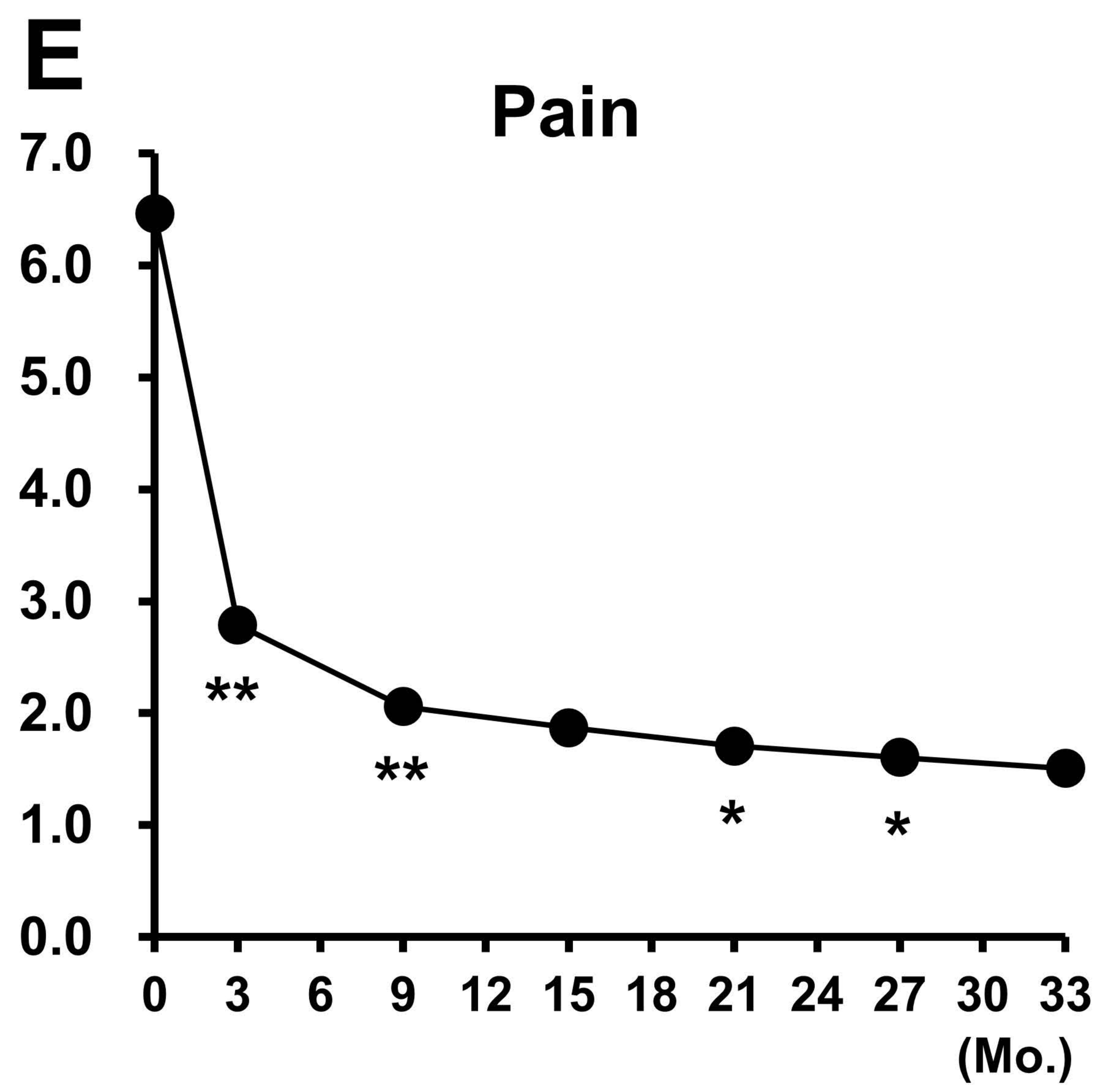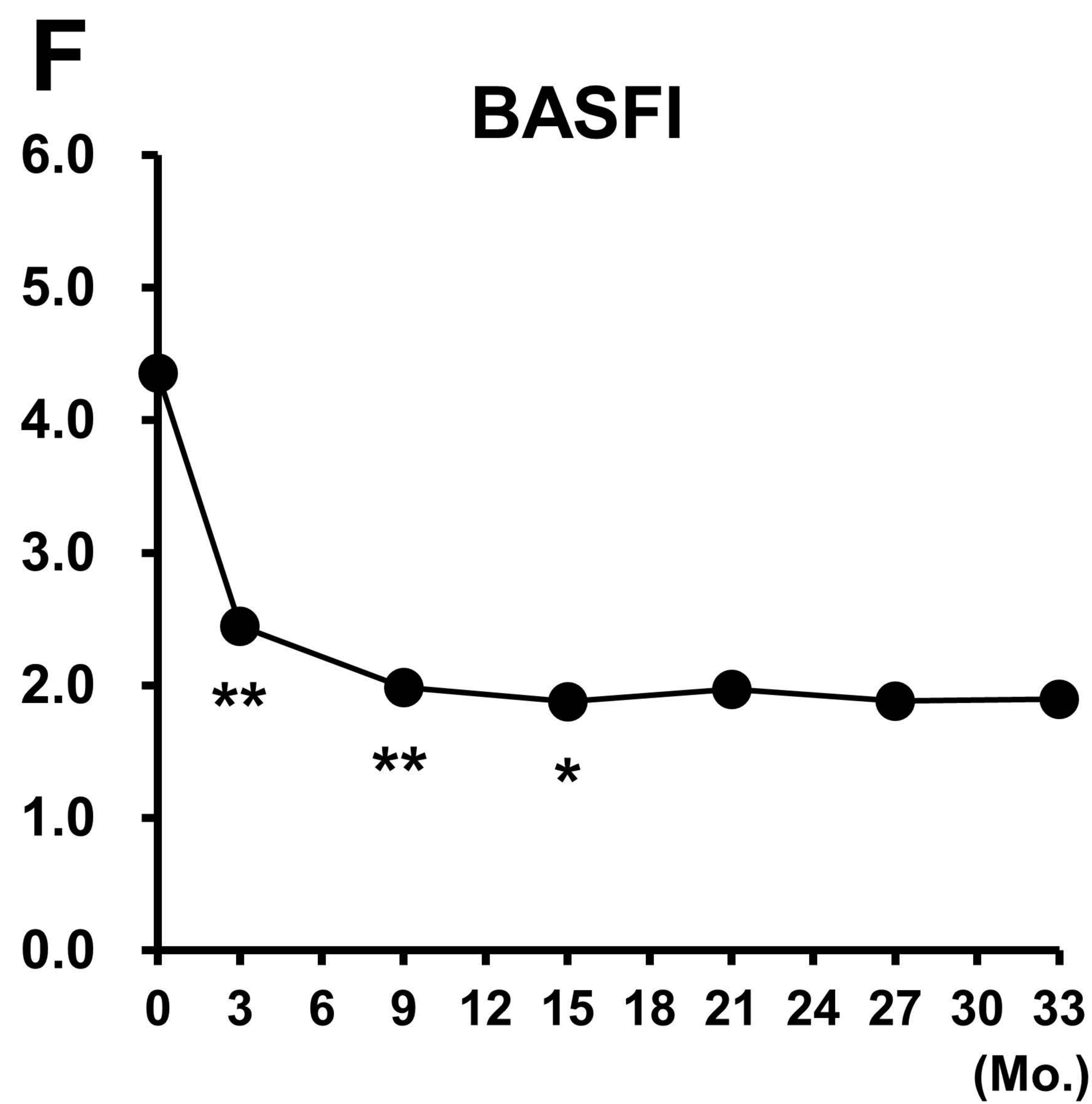

Supplement: Supplementary file 1 [file jcm-10-04279-s001.zip › Figure S4-J Clin Med.pdf]

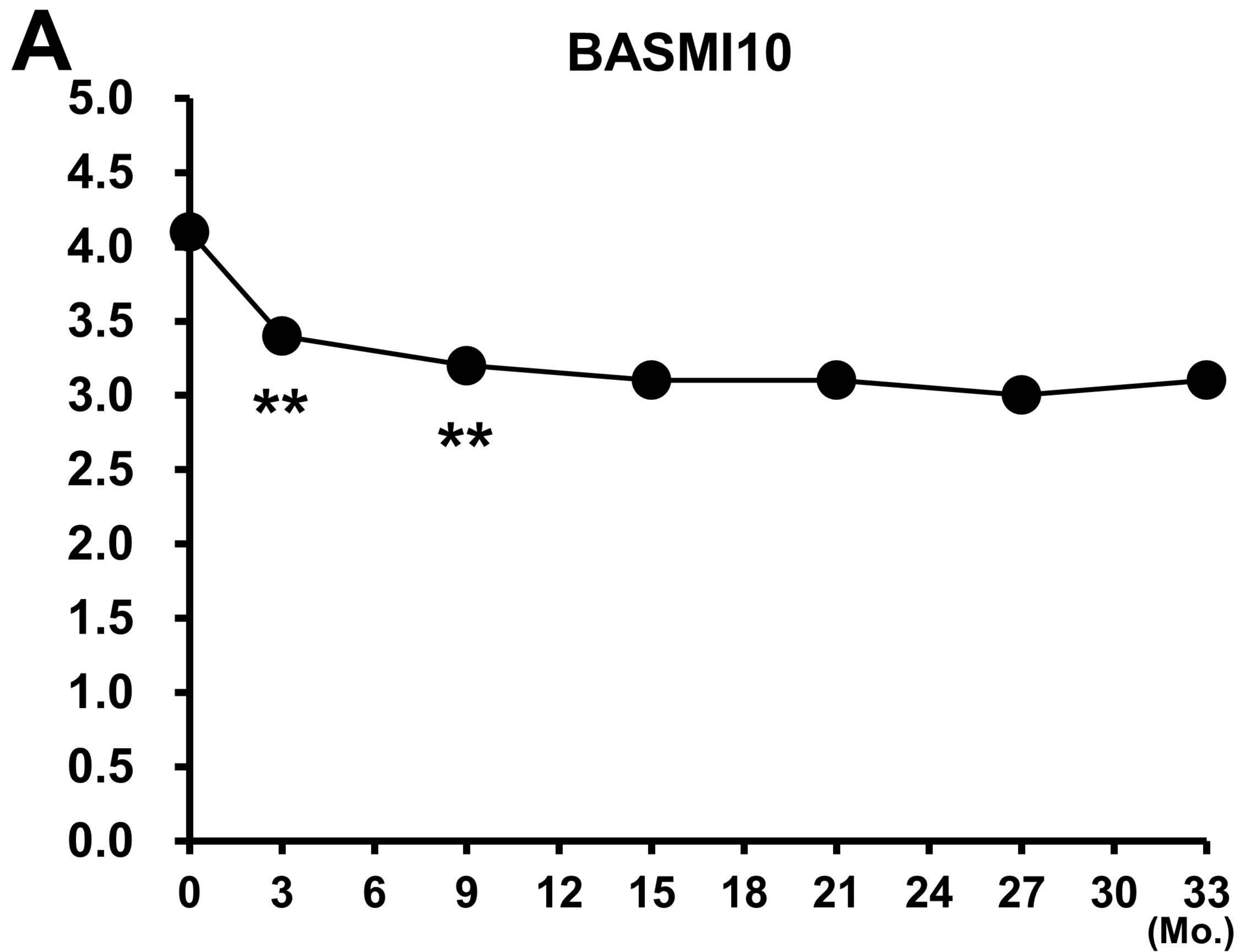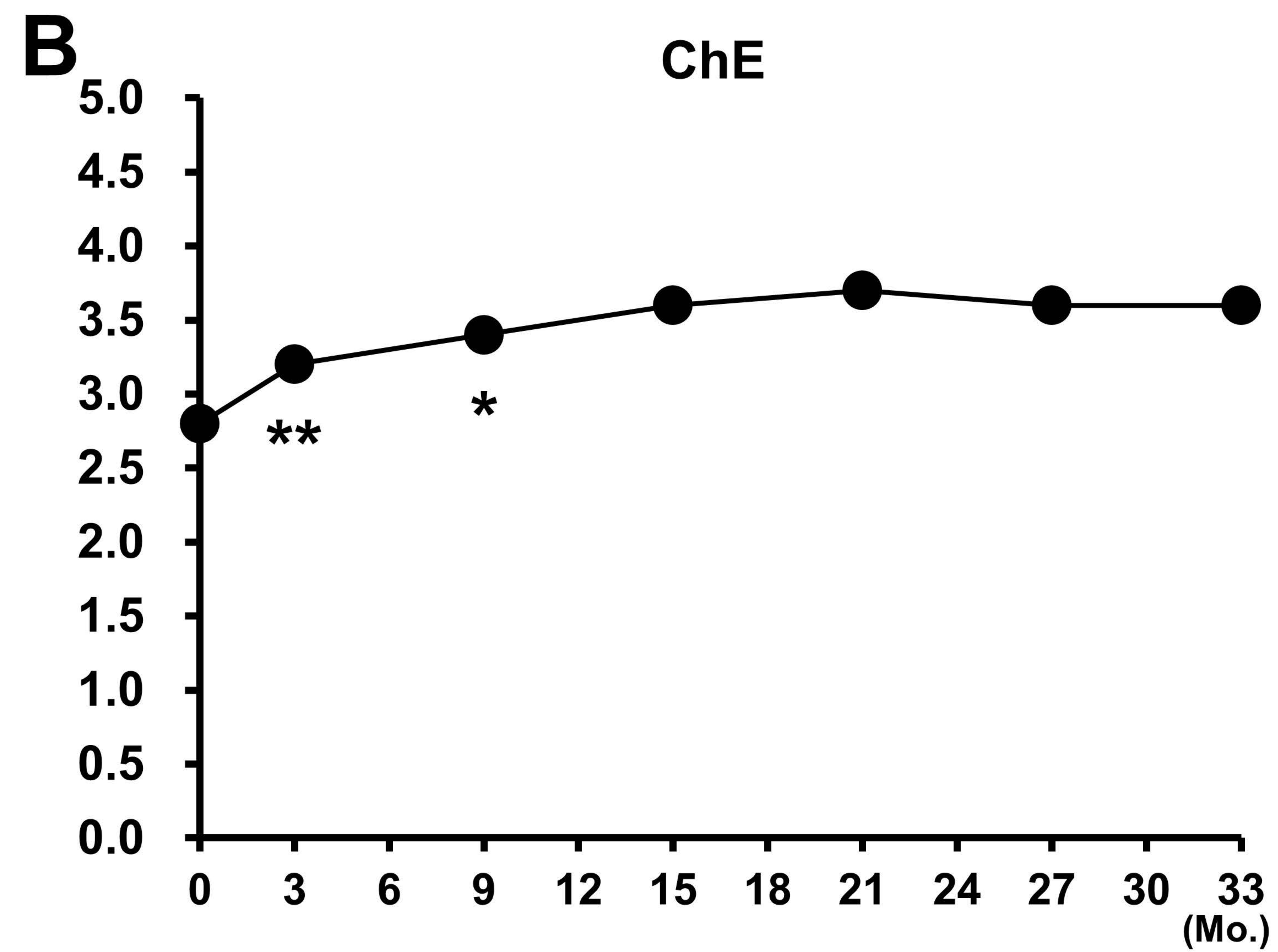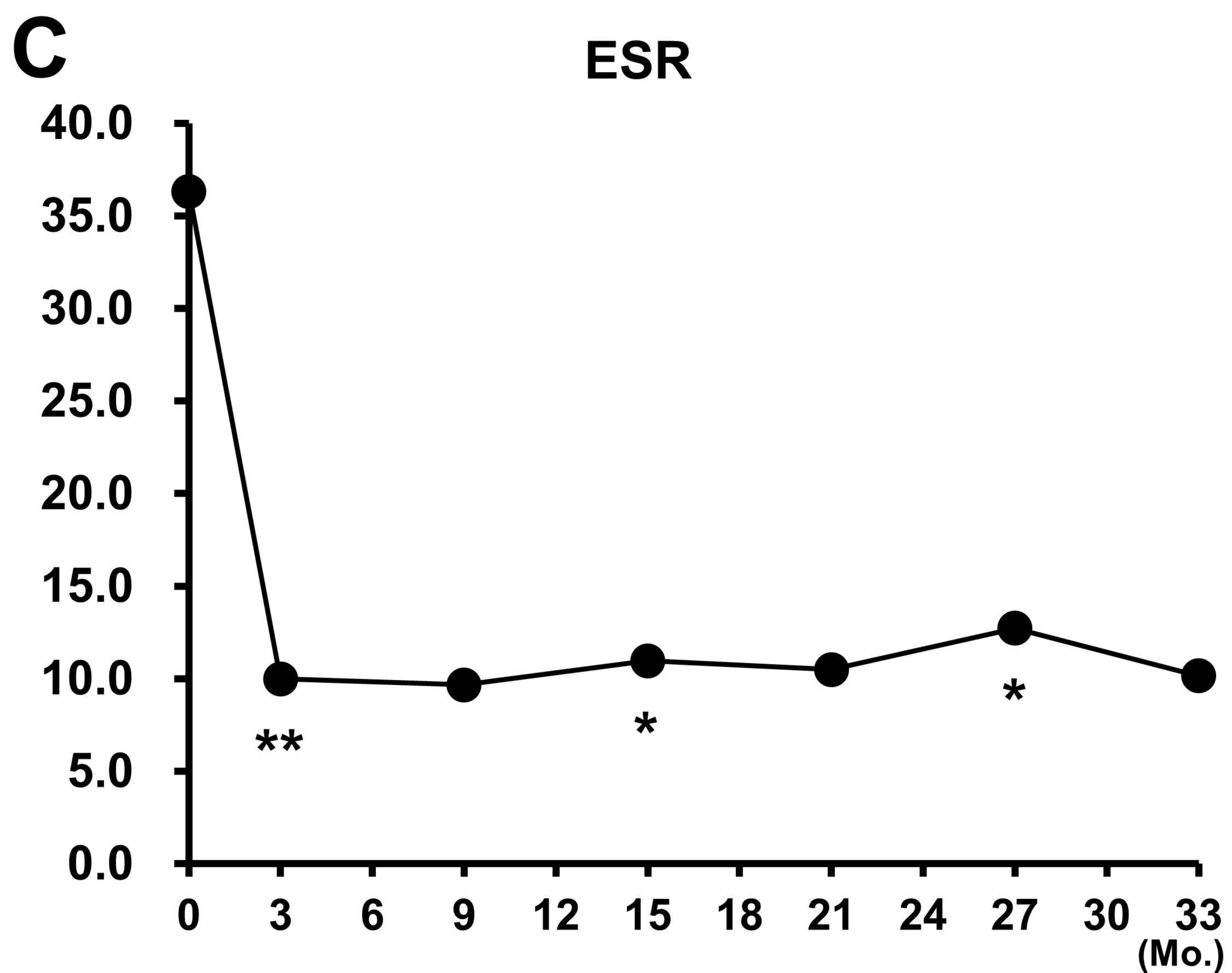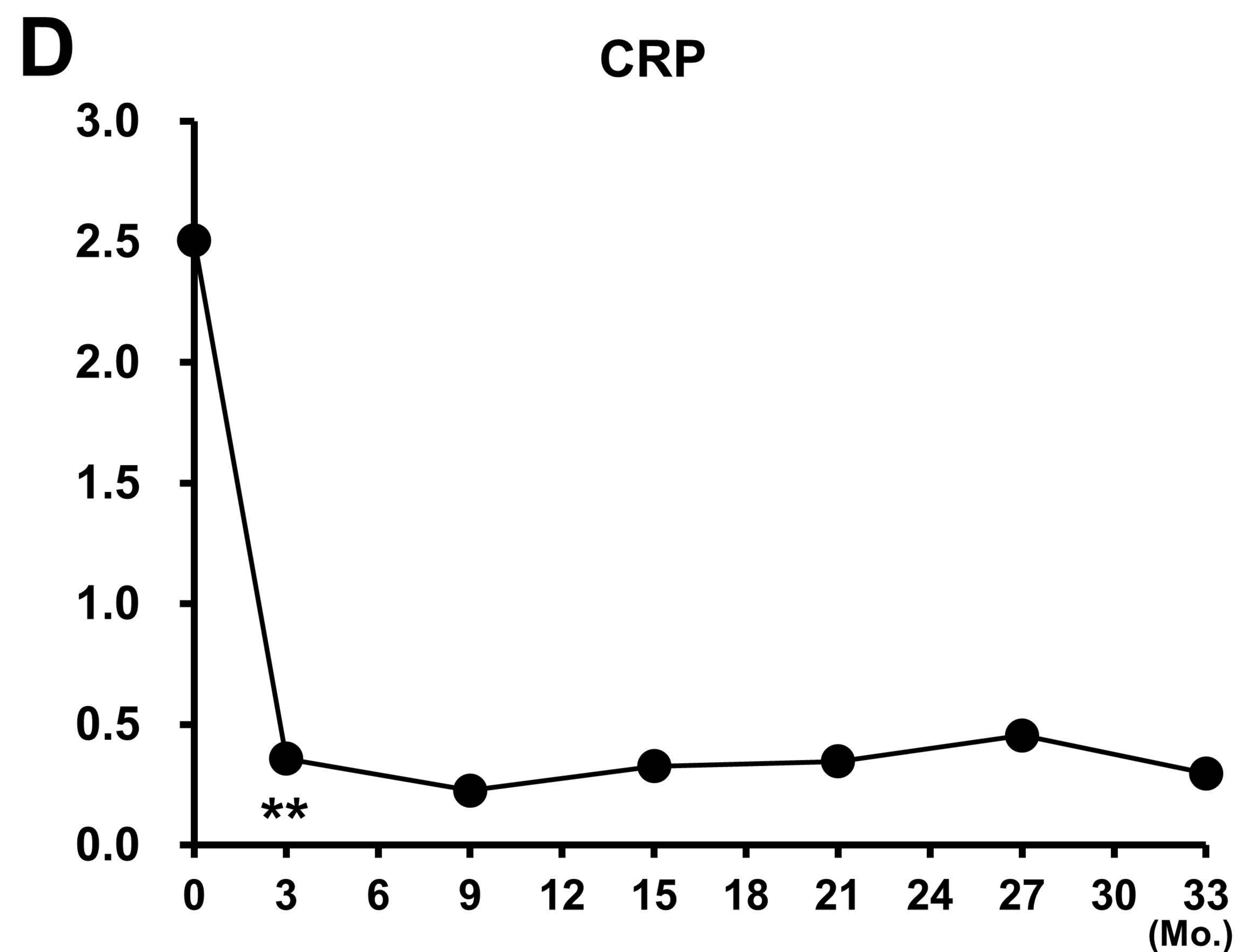

Supplement: Supplementary file 1 [file jcm-10-04279-s001.zip › Figure S5-J Clin Med.pdf]
